# Temporal Variability in the Incidence and Risk Factors for Pharyngocutaneous Fistula Development after Total Laryngectomy

**DOI:** 10.3390/cancers16203486

**Published:** 2024-10-15

**Authors:** Robert Šifrer, Maja Dolenc, Sara Bitenc Zore, Simon Fugina, Luka Jesenko, Primož Strojan

**Affiliations:** 1 Department of Otorhinolaryngology and Cervicofacial Surgery, University Medical Centre Ljubljana, Zaloška 2, 1000 Ljubljana, Slovenia; 2 Faculty of Medicine, University of Ljubljana, Vrazov trg 2, 1000 Ljubljana, Slovenia; 3 Department of Radiation Oncology, Institute of Oncology, Zaloška 2, 1000 Ljubljana, Slovenia; pstrojan@onko-i.si

**Keywords:** total laryngectomy, fistula, incidence, fluctuation, periods, surgical wound infection, head and neck cancer, timely management

## Abstract

**Simple Summary:**

The pharyngocutaneous fistula (PCF) is a pathologic canal connecting the pharyngeal lumen with the skin of the neck occurring after a total laryngectomy (TLE), the removal of the entire larynx. The incidence of PCF ranges from 0% to 80%. Our study aimed to identify the temporal changes in PCF incidence over an extended period and determine the risk factors for increases in the PCF rate. By reviewing patient data from 2004 to 2022, we discovered a total incidence of 26.7%. This study revealed four high-incidence periods, averaging 37.61%, each followed by lower-incidence periods of 19.38%. The surgical wound infection and a history of head and neck cancer along with its related treatments were identified as independent risk factors during most of the high-incidence periods. The continuous monitoring of patients following TLE can assist providers in the better prediction and, consequently, timely management of PCF, ultimately improving patient outcomes after TLE.

**Abstract:**

**Background:** This study aimed to analyse the variability in the incidence of the pharyngocutaneous fistula (PCF), the most common complication following a total laryngectomy (TLE), and to identify the underlying causes for fluctuations in incidence rates. **Methods:** In the retrospective study, the annual PCF incidence data and comprehensive clinicopathologic data from 540 patients who underwent TLE between January 2004 and December 2022 were reviewed. Distinct peri ods of both high and low PCF incidence were identified. Within these periods, patients were categorized into groups with PCF (study groups) and without it (control groups). These groups were statistically compared based on potential risk factors for PCF development. The high-incidence periods were specially analysed for recurring risk factors and the corresponding corrective measures were reviewed. **Results:** The analysis revealed four high-incidence periods with an overall PCF incidence of 37.61%, along with three low-incidence periods in between with an overall incidence of 19.38%. Surgical wound infection (SWI) and a history of head and neck cancer alongside their related treatments were repeatedly identified as independent risk factors during high-incidence periods, with SWI being the most consistent predictor of PCF development. **Conclusions:** Continuous monitoring of PCF incidence is crucial, as it allows for the identification of emerging risk factors and the immediate implementation of corrective measures to mitigate these newly identified risk factors.

## 1. Introduction

The incidence and risk factors for the development of pharyngo-cutaneous fistula (PCF) following a total laryngectomy (TLE) have already been extensively discussed in the literature. A wide range of PCF incidence rates have been reported, ranging from 0% [1] to 80% [2]. According to recent meta-analyses by Kim et al. [3] and Wang et al. [4], the incidence of PCF is 21.69% and 21%, respectively. In general, it decreases (8–25%) after primary TLE [5,6,7,8,9,10] and increases after salvage TLE (14–57%) [5,6,9,10,11,12]. The results of our previous analysis on 422 patients with a PCF rate of 23.9% (20.8% after primary TLE and 32.7% after salvage TLE) were in line with the literature’s data [13].

There is a plethora of identified risk factors for the development of PCF. However, risk factors differ significantly from article to article. According to recent meta-analyses, the main risk factors include an age exceeding 60, a history of smoking, chronic obstructive pulmonary disease, coronary atherosclerosis, diabetes mellitus, previous radiotherapy (RT), a previous tracheostomy, a low preoperative and postoperative serum albumin and haemoglobin level, a primary tumour stages T3–4, a supraglottic tumour site, an additional pharyngectomy, salvage TLE, and a primary tracheoesophageal puncture [3,4]. In light of our previous study, the independent risk factors for the development of PCF following a primary TLE are surgical wound infection (SWI) and the invasion of piriform sinus, whereas in salvage TLE these are SWI and a dose of previous irradiation [13].

The incidence and risk factors for PCF have been researched and followed up regularly by the University Department of Otorhinolaryngology and Cervicofacial Surgery in Ljubljana since 2004. In our experience, the PCF rate was not constant but varied considerably over time. The aim of this study was to analyse the variability of the incidences in a year-to-year setting and discover the causes for each individual rise (i.e., spike) and fall in the incidence of PCF. This way, we could discover the actual and newly emerging causes for the rise of the incidence of PCF. In our opinion, being aware of the most recent causes for PCF could result in the most appropriate and efficient remedial measures what would lead to the reduction of PCF rate.

## 2. Materials and Methods

The charts of patients treated for head and neck cancer (HNC) with TLE between January 2004 and December 2022 at the University Department of Otorhinolaryngology and Cervicofacial Surgery in Ljubljana were reviewed. The data associated with the patient, disease, treatment, and post-operative course were collected and are shown in Table 1.

Initially, the incidence of PCF was calculated for the whole cohort, i.e., the total incidence, and then for each year of the study separately, i.e., the annual incidences. The latter were expected to be either higher or lower than the former. The consecutive years of TLE with higher annual incidences than the total incidence were grouped together as were the ones with lower incidences (compared to the total incidence). In this manner, precisely defined periods of high annual incidences of PCF and periods of low annual incidences were obtained.

Within each period, the patients were categorised into groups with PCF (study groups) and groups without it (control groups). Moreover, the groups within individual periods of high and low incidences were statistically compared according to the potential risk factors for PCF development that are presented in Table 1. This way, the statistically significant risk factors were determined for each particular period. Emphasis was placed on those risk factors that are amenable to preventive actions by the surgeon.

The statistical analyses were performed using the IBMI SPSS Statistics version 25 (Chicago, IL, USA). For comparative analyses, the Chi square test, Fisher’s exact test, *t*-test, and Mann–Whitney U test were used. To determine the independent predictive values for the PCF formation of different potential risk factors in periods of high incidences, a binary logistic regression analysis was undertaken. Only factors that proved to be significant in the univariate analysis were included. All statistical tests were two-sided and *p*-values below 0.05 were considered statistically significant.

The study was conducted according to the guidelines of the Declaration of Helsinki, and approved by the National Medical Ethics Committee (protocol code 0120-338/2021/16) on 7 January 2022.

## 3. Results

A total of 540 patients were included in this study. The primary tumour sites included the larynx in 355 patients (65.7%), the hypopharynx in 170 patients (31.5%), the oropharynx in 13 patients (2.4%), and the oral cavity and thyroid gland in 1 patient (0.2%) each. The mean age of the patients was 63.6 years (with a standard deviation of 9.8 years) and 493 (91.3%) of them were male.

TNM staging according to the eighth edition of the TNM classification of malignant tumours was used [14]. The primary tumour classification was pT1 in 17 patients (3.2%), pT2 in 64 patients (12.0%), pT3 in 264 patients (49.4%), and pT4a in 189 patients (35.4%). The neck was staged pN0 in 269 patients (50.1%), pN1 in 48 patients (8.9%), pN2a in 29 patients (5.4%), pN2b in 39 patients (7.3%), pN2c in 25 patients (4.7%), and pN3b in 127 patients (23.6%). The T and N stages remained unknown in 6 and 3 patients, respectively. All patients were free from distant metastases.

In two patients, clinical data regarding PCF could not be retrieved due to insufficient medical documentation and were excluded from this study. The PCF developed in 144 of 538 patients (26.8%, total PCF incidence). The total incidence in the primary TLE group (i.e., without previous (C)RT to the larynx) was 22.2% (88/396), while in the salvage TLE group, it was 39.7% (56/141) (*p* < 0.001, Chi square test). 

**Figure 1:**
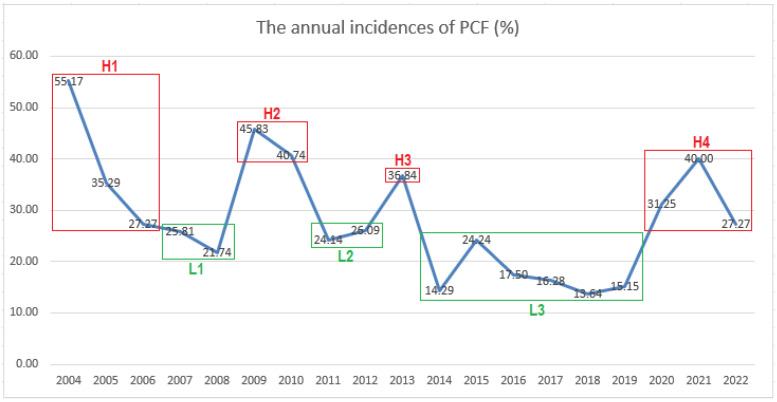
The annual incidences in relation to the year of TLE. Periods of high incidences (H1–H4) are marked in red and periods of low incidences (L1–L3) are marked in green.

The annual incidences and their relation to the total incidence are depicted in Table 2. Based on the consecutive years of either high or low annual incidence of PCF in relation to the total incidence, seven periods were defined. Notably, there were four periods of high incidence (H1–H4) and three periods of low incidence (L1–L3), as illustrated in Figure 1 and Table 2. Overall, nine years were distributed among the high-incidence periods and ten years among the low-incidence periods. The mean rates of PCF in the H1, H2, H3, and H4 periods were 39.24%, 43.14%, 36.84%, and 31.88%, and in the L1, L2, and L3 periods, 24.07%, 25.00%, and 16.82%, respectively. The annual incidence in the periods of high PCF rates ranged from 27.27% to 55.17%, while periods of low rates ranged from 13.64% to 26.09% (Figure 1, Table 2). The overall rate of PCF in high and low incidence periods was 37.61% (82/218) and 19.38% (62/320), respectively.

Periods of high (H1–H4) and low (L1–L3) incidences of PCF and the statistically significant risk factors for the development of PCF as determined by univariate analysis are reported in Table 3.

The results of a binary logistic regression analysis of the factors proved significant upon univariate analysis for high-incidence periods are presented in Table 4.

Due to an unexpectedly high incidence of PCF in 2004 (55.17%), which prompted us to systematically track the incidence of PCF in our department from then on, the data for that year were analysed separately. The risk factors for the formation of PCF in 2004 by univariate analysis were SWI (81.3% vs. 23.1%, *p* = 0.003), transfusion (37.5% vs. 0%, *p* = 0.020), and the duration of surgery (3.7 h vs. 2.6 h, *p* = 0.013). A binary logistic regression revealed that the independent risk factor for the development of PCF in 2004 was SWI (odds ratio = 14.44, 95% CI = 2.39–87.40, *p* = 0.004).

The polyglycolic acid sutures were introduced for closure of neopharyngeal canal in 2009 and had been in use until the end of 2010, in other words, only in H2. As the comparison within H2 would not give any results, we determined the impact of polyglycolic sutures by combining H2 to the previous period L1. Both periods combined, the polyglycolic acid sutures appeared as statistically significant risk factor for PCF (62.9% vs. 41.4%, *p* = 0.038).

## 4. Discussion

The aim of our study was to determine the temporal pattern of change in the incidence of PCF after TLE over a long period of time and to analyse the patterns of this development. The total incidence of PCF in our series was 26.7%; specifically, 22.2% after primary TLE and 39.7% after salvage TLE, which are within the expected ranges given the literature’s results [5,6,7,8,9,10,11,12]. The incidence of PCF fluctuated considerably from 13.64% (in 2018) to 55.17% (in 2004). After analysis of the annual PCF incidence rates, we identified four high-incidence periods (H1–H4) with an overall incidence of 37.61% and three low-incidence periods (L1–L3) with an overall incidence of 19.38% (Figure 1 and Table 2).

The appearance of new and previously non-existent factors as well as the initiation of practices that impacted known risk factors or clinical routines resulted in variations in PCF incidence. This is a crucial finding of our study. The dynamics of the occurrence of PCF, the identified causes, and the remedial measures are all shown in Table 5. 

The statistically significant risk factors for PCF that repeatedly appeared in this high-incidence periods are of particular importance. In binary logistic regression, SWI and previous HNC with related treatment(s) were determined as independent risk factors for PCF in three periods of high incidence—H1, H2, and H4 (Table 4). On the other hand, risk factors associated with patient malnutrition (low protein levels, weight loss, alcohol abuse) appeared statistically significant in two periods of high incidence (H3 and H4) but in the univariate analysis only (Table 3). 

SWI was the most consistent risk factor for the development of PCF throughout our study. During the surgery, the pharynx is opened and the sterile environment of the neck is exposed to the upper aerodigestive tract’s bacterial flora [15,16] classifying the surgical wound after TLE as clean-contaminated. As such, it is prone to SWI, which occurred in 10% [17] to 44% [18] of cases. For the purpose of this study, SWI was defined as a grade three adverse effect according to Tabet and Johnson’s classification [15], characterized by erythema, oedema and the induration of the suture line and surrounding skin. In the case of TLE, SWI can have an exceedingly detrimental influence which may include the dehiscence of the pharyngeal suture line leading to the formation of PCF [13,19]. 

To reduce the adverse influence of SWI, several measures to improve aseptic working methods were implemented throughout the studied period. At first, the surgeons were discouraged from inappropriate prolongation of the antibiotic prophylaxis [16,19] and it was suggested that they provide daily care of the surgical wound personally using bacteriocidic alcohol-based solutions. Secondly, clindamycin, with its insufficient Gram-negative coverage and bacteriostatic (but not bacteriocidic) mechanism of action [20], was replaced by amoxicillin-clavulanic acid (Table 5).

The influence of previous HNC on PCF formation and the related interventions have been extensively discussed in the literature [3,4,5,13,21,22,23,24,25]. Boscolo-Rizzo found no relationship between the history of previous HNC and PCF [21]. However, RT, RCT, and surgical interventions for previous HNC leave sequelae in the tissues of the affected area. 

At first, RT and particularly CRT (increasing both local effects of RT on HNC and toxic side-effects on the surrounding tissues, [6,12]) diminish tissue perfusion by obliterative endarteritis, hypoxia, impaired leukocyte migration and fibrosis on the microscopic level [5,21]. However, the literature has been inconsistent regarding its influence on PCF formation. Recent studies support the idea of an increased incidence of PCF in irradiated patients [3,4,13], whereas older ones do not [21,23,24]. Our current results are in agreement with the former. Nevertheless, if previously irradiated patients develop PCF, these are often extensive, persistent, intractable, and, according to Weinberger, of higher grades usually requiring surgical intervention(s) [5,23,25,26,27]. Secondly, providing haemostasis during a previous surgery (ligation, clipping of vessels) results in the so-called vessel-depleted neck on a macroscopic level. Within the framework of the herein-described consequences, the impaired vascularisation of the neck is the final result [28,29,30]. Therein, the diminished tissue perfusion and oxygenation are unavoidable and negatively impact the healing capabilities of the pharynx after subsequent salvage TLE [24].

Blood transfusions and increased erythrocyte sedimentation rate after TLE were also found independently related to PCF, but only in single periods of high incidence—H1 and H3, respectively. The need for transfusion after TLE is the consequence of major blood loss. It reduces oxygenation and nutrition disturbing the healing of surgical wounds which is highly demanding in terms of metabolism. Blood loss could be the result of a substandard surgical technique on the one hand, and coagulation disorders on the other. An atraumatic, flawless surgical technique is probably the most important factor in terms of reducing PCF formation [6]. To prevent the need for transfusion, we strove for better medical and anaesthesiological preparation of patients for surgery and reminded surgeons of the appropriate surgical technique, especially in terms of the meticulous pharyngeal closure and inversion of the mucosa within the lumen of the neopharynx. Moreover, the TLEs have been performed by experienced personnel since. Beginners were allowed to perform the most basic, simple steps of TLE, e.g., skin incisions, subplatysmal flap elevation, and the initial approach to the larynx. Any more complicated or demanding steps, such as planning the excision of the cancer, the removal of the larynx, frozen section analysis, pharyngeal closure, and reconstructions have been exclusively executed by experienced surgeons (Table 5).

The term inflammatory parameter represents a common umbrella under which erythrocyte sedimentation rate, leukocyte count and C-reactive protein can be collected. Their levels are typically raised in inflammation. As SWI is the strongest risk factor for the development of PCF in our series, the increase in the levels of inflammatory parameters in the context of PCF is a logical expectation. 

Other risk factors as well as our corresponding corrective measures can be found in Table 5. As important steps in our effort to reduce the incidence of PCFs, we have considered the implementation of a new suturing technique for the neopharynx (running sutures), the replacement of the current suturing material (polyglactin 910 instead of the polyglycolic acid sutures), and counselling with a clinical nutritionist before surgery. 

Taking into account the multitude of studies in the literature including systemic reviews and meta-analyses [3,4,6,9,31,32,33], the data on incidence and significance of risk factors are extremely variable explaining the difficulty in reaching a consensus regarding the most important risk factors for the development of the PCF [34]. This could point to the usefulness, if not the uselessness, of particular meta-analyses for particular clinicians or departments. On the basis of our results, we concluded that the incidence of PCF is not constant but can fluctuate considerably (Figure 1, Table 2). In addition, the significance of the individual potential risk factors may vary over time (Table 3 and Table 4). We assert that each department is an individual entity with its own issues and, consequently, its own (annual) incidence(s) and unique set of risk factors for PCF formation alongside other complications. 

From our point of view, in terms of the follow-up for PCF incidence, it is of utmost importance to identify new risk factors and take corrective measures to eliminate them. However, some factors can be influenced, while others cannot. The former are undoubtedly of fundamental importance. It is in this way that a surgeon can reduce PCF incidence. Unfortunately, once one risk factor has been eliminated, another may later emerge, so constant monitoring is also of paramount importance.

Therefore, we suggest that each department should track its own incidence of PCFs and the risk factors involved in their development. Having identified them, the department should react properly and promptly to eliminate them. In this sense, an ongoing and up-to-date analysis of complications on an annual basis would be the best option. To further illustrate this, a study on the incidence and risk factors of each year should be completed every December, so that its results can be implemented from January onwards the following year.

Our study has some limitations, primarily due to the retrospective design and its inherent constraints related to the reliability and completeness of the collected data. The present series includes data and experience from a single centre, so further studies with a comparable research methodology are warranted.

## 5. Conclusions

PCF continues to be a fairly common complication following TLE. Our experience with the overall PCF incidence of 26.8% is consistent with the results of other studies. However, in the various periods, the incidence of PCF in our patients varied considerably, between 19.38% and 37.63%. Additionally, some of the risk factors found (previous therapies, SWI) were ubiquitous, i.e., they occurred in the majority of periods with high PCF rates while others were specific and characteristic only for one individual period (transfusion, erythrocyte sedimentation rate). It is therefore advisable to keep a constant eye on such risk factors, particularly those that can be prevented or, mitigated at the very least.

## Figures and Tables

**Table 1:** Potential risk factors for the development of PCF; HNC = head and neck cancer, RT = radiotherapy, CRT = chemoradiotherapy, (C)RT = (chemo)radiotherapy, ASA = American Society of Anaesthesiologists, TLE = total laryngectomy, HPh = hypopharynx, OPh = oropharynx, ECS = extracapsular spread, PhE = pharyngectomy, SCAIF = supraclavicular artery island flap, CRP = C reactive protein, ESR = erythrocyte sedimentation rate.

Potential Risk Factors Associated with the Patient	Potential Risk Factors Associated with the Disease	Potential Risk Factors Associated with Surgery	Potential Risk Factors Associated with the Post-Operative Period
Age	Primary site	Dyspnoea management	Surgical wound infection Yes/no
Sex	Larynx	Tracheostomy	Beginning
Comorbidities	Hypopharynx	Debulking	Postop. lab. values
Diabetes	Oropharynx	Primary tumour surgery	Haemoglobin
Cardiovascular	Oral cavity	LE + partial PhE	Albumin
Pulmonary	Thyroid gland	LE + total PhE	Proteins
Gastrointestinal	Subsite	Neck dissection	CRP
Central nervous system	Glottis	Unilateral	Leukocytes
Hypercholesterolemia	Supraglolttis	Bilateral	ESR
Weight loss	Subglottis	Surgical margin status	Difference (lab. values)
Amount of kg loss	Larynx-median line	Suture material	Haemoglobin
Abuse of	Larynx-bilaterally	Suturing technique	Albumin
Tobacco	Piriform sinus	Reconstruction	Proteins
Alcohol	Retrocricoid area	Epiglottoplasty	
Preop. laboratory values	Post. wall of HPh	Major reconstruction	
Haemoglobin	HPh-median line	Type of flap	
Albumin	HPh-bilaterally	Pectoralis major	
Proteins	Oropharynx	SCAIF	
History of cancer	OPh-median line	Radial forearm	
Any site	OPh-bilaterally	Anterolateral thigh	
No HNC	Histology	Gastric pull up	
HNC	Type	Duration of surgery	
Site of HNC	Grade	Surgeon	
Treatm. of previous HNC	Stage	11 surgeons	
RT	T	Blood transfusion	
CRT	N		
(C)RT	M		
Dose of RT	No. of positive nodes		
Interval (RT—TLE)	ECS		
Surgery	Synchronous tumour		
Type of surgery	Site		
Interval (surgery—TLE)			
Surgery or RT or CRT			
ASA score			

**Table 2:** The annual incidences of PCF and their relation to the total incidence as well as the definition of high (H1, H2, H3, H4) and low incidence periods (L1, L2, L3) of PCF. TLE = total laryngectomy, PCF = pharyngocutaneous fistula.

Year of the Study	Patients with PCF	All Patients with TLE	Annual Incidence (%)	Relation of Annual Incidences to Total Incidence	Name of Period
2004	16	29	55.17	High	H1
2005	6	17	35.29	High	
2006	9	33	27.27	High	
2007	8	31	25.81	Low	L1
2008	5	23	21.74	Low	
2009	11	24	45.83	High	H2
2010	11	27	40.74	High	
2011	7	29	24.14	Low	L2
2012	6	23	26.09	Low	
2013	7	19	36.84	High	H3
2014	3	21	14.29	Low	L3
2015	8	33	24.24	Low	
2016	7	40	17.50	Low	
2017	7	43	16.28	Low	
2018	6	44	13.64	Low	
2019	5	33	15.15	Low	
2020	10	32	31.25	High	H4
2021	6	15	40.00	High	
2022	6	22	27.27	High	

**Table 3:** Statistically significant risk factors for PCF by univariate analysis and the influence of surgical techniques (single, continuous sutures for pharyngeal repair) on PCF in the periods of high incidences of PCF (a: *t*-test, b: Chi square test, c: Fisher’s exact test, d: Mann–Whitney U test, e: Not statistically significant). RT = radiotherapy, CRT = chemoradiotherapy, (C)RT = (chemo)radiotherapy, PM = pectoralis major myocutaneous flap, CNS = central nervous system, PCF = pharyngocutaneous fistula, HPh = hypopharynx, CRP = C reactive protein, ESR = erythrocyte sedimentation rate.

Period	Years	Risk Factor	Overall, during This Period	Group with PCF during This Period	Group without PCF during This Period	*p* Value
H1	2004–2006	RT	23 (29.5%)	13 (41.9%)	10 (21.3%)	0.05 ^b^
		Transfusion	13 (17.1%)	9 (31.0%)	4 (8.5%)	0.025 ^c^
		Surgical wound infection	30 (39%)	23 (76.7%)	7 (14.9%)	<0.001 ^b^
L1	2007–2008	Dose of RT	63	70	63	0.026 ^d^
		Cardio-vascular disease	20 (37.0%)	9 (69.2%)	11 (26.8%)	0.009 ^c^
		Invasion of piriform sinus	19 (35.2%)	8 (61.5%)	11 (26.8%)	0.043 ^c^
		Invasion of retrocricoid area	12 (22.2%)	6 (46.2%)	6 (14.6%	0.027 ^c^
		Invasion of oropharynx	12 (22.2%)	6 (46.2%)	6 (14.6%	0.027 ^c^
		R_+_ resection	2 (3.9%)	2 (18.4%)	0	0.043 ^c^
		Type of flap—PM	2 (3.7%)	2 (15.4%)	0	0.033 ^b^
		Type of flap—radial forearm	1 (1.9%)	0	1 (2.4%)	
		Surgical wound infection	17 (31.5%)	11 (84.6%)	6 (14.6%)	<0.001 ^b^
H2	2009–2010	Previous head and neck cancer	19 (37.3%)	12 (54.5%)	7 (24.1%)	0.026 ^b^
		Invasion of HPh bilaterally	4 (7.8%)	4 (18.2%)	0	0.029 ^c^
		Surgical wound infection	22 (43.1%)	16 (72.2%)	6 (20.7%)	<0.001 ^b^
		Start of surgical wound inf.	7	6	10	0.04 ^a^
L2	2011–2012	Age	64.9	59.7 years	66.6 years	0.022 ^a^
		Duration of surgery	4.1 h	4.9 h	3.8 h	0.046 ^a^
		Protein level before surgery	69.41	66.45	70.57	0.043 ^a^
		CNS disease	10 (19.5%)	6 (46.2%)	4 (10.3%)	0.010 ^c^
		Invasion of post. wall of HPh	3 (5.8%)	3 (23.1%)	0	0.013 ^c^
		Type of flap—PM	5 (9.6%)	4 (30.8%)	1 (2.6%)	0.011 ^c^
		Surgical wound infection	18 (34.6%)	11 (84.6%)	7 (17.9%)	<0.001 ^c^
H3	2013	Duration of surgery	5.3 h	6.8 h	4.3 h	0.012 ^a^
		Protein level after surgery	53.53	55.33	50.71	0.033 ^a^
		Protein level difference	13.28	18.17	10.83	0.013 ^a^
		Cardio-vascular disease	12 (63.2%)	7 (100%)	5 (41.7%)	0.017 ^b^
		Alcohol abuse	8 (47.1%)	5 (83.3%)	3 (27.3%)	0.050 ^b^
		Invasion of oropharynx	5 (26.3%)	4 (57.1%)	1 (8.3%)	0.038 ^c^
		Type of flap—epiglottoplasty	5 (26.3%)	4 (57.1%)	1 (8.3%)	0.038 ^c^
		ESR after TLE	68.68	88.71	57.00	0.014 ^a^
L3	2014–2019	Duration of surgery	5.4 h	6.1 h	5.2 h	0.017 ^a^
		Invasion of post. wall of HPh	18 (8.4%)	6 (16.7%)	12 (6.7%)	0.05 ^b^
		Synchronous cancer	15 (7.0%)	6 (16.7%)	12 (6.7%)	0.013 ^b^
		Reconstruction	35 (16.4%)	11 (30.6%)	24 (13.5%)	0.012 ^b^
		Surgical wound infection	46 (21.5%)	24 (66.7%)	22 (12.4%)	<0.001 ^b^
		Leukocytes after TLE	12.8	14.1	12.6	0.041 ^a^
		CRP after TLE	115	143	110	0.002 ^a^
		Single sutures	84 (39.3%)	19 (52.8%)	65 (36.5%)	0.068 ^b,e^
		Continuous sutures	100 (60.7%)	17 (47.2%)	113 (63.5%)	
H4	2020–2022	Protein level before surgery	66.25	64.09	67.28	0.030 ^a^
		No. of kg loss	6.12	9.67	4	0.011 ^a^
		Previous cancer—any	22 (31.9%)	11 (50%)	11 (23.4%)	0.027 ^b^
		Previous head and& neck cancer	16 (23.2%)	9 (40.9%)	7 (14.9%)	0.017 ^b^
		CRT	6 (8.7%)	5 (22.7%)	1 (2.1%)	0.011 ^c^
		(C)RT	13 (18.8%)	10 (45.5%)	3 (6.4%)	<0.001 ^c^
		Surgery + (C)RT	5 (7.2%)	4 (18.2%)	1 (2.1%)	0.033 ^c^
		Surgical wound infection	22 (32.4%)	19 (86.4%)	3 (6.5%9	<0.001 ^c^
		Leukocytes after TLE	13.4	15.2	12.5	0.023 ^a^
		CRP after TLE	136.4	195	109	<0.001 ^a^

**Table 4:** A binary logistic model for patients of high-incidence periods. HNC = head and neck cancer, ESR = erythrocyte sedimentation rate.

Period	Years	Risk Factor	Odds Ratio	95% CI	*p* Value
H1	2004–2006	Radiotherapy	5.69	1.25–25.87	0.024
		Transfusion	8.38	1.38–50.79	0.021
		Surgical wound infection	26.28	6.27–110.19	<0.001
H2	2009–2010	Previous HNC	5.39	1.20–24.22	0.028
		Surgical wound infection	13.00	2.99–56.61	0.001
H3	2013	ESR after TLE	1.06	1.00–1.13	0.048
H4	2020–2022	Chemoradiotherapy	85.40	3.87–1886.96	0.005
		Surgical wound infection	226.10	21.99–2325.05	<0.001

**Table 5:** The reasons for the change in PCF incidence and corrective measures to reduce it and the types of evidence supporting the corrective measures. PCF = pharyngocutaneous fistula, CM = corrective measure; the evidence of corrective remeasures: ^uni^ = univariate analysis, ^bin^ = binary logistic regression.

Year/ Period	Cause of the Increase in PCF Incidence	Consequence	Corrective Measures	Year of CM	Consequence of Corrective Measure
	Un-researched causes (before 2004)	Extremely high rate of PCF	Incidence and risk factor tracking		
	Surgical wound infection^bin^	Incidence 55.17%	Daily wound care by the surgeon		
			Improved aseptic work methods		Decrease in 2005, 2006
2004	Transfusion^uni^	Incidence 55.17%	Better medical and anaesthesiological preparation	2005	L1
			Improvement of surgical techniques		
	Long duration of surgery^uni^	Incidence 55.17%	Shortening		
	Introduction of new sutures (polyglycolic acid)^uni^	Rise in H2	Removal of the polyglycolic acid sutures		
			Re-introduction of polyglactin 910		
H2	Surgical wound infection^bin^	Rise in H2	Replacing clindamycin with amoxicillin—clavul.	2011	L2
			Dissuaded from prolonged antibiot. prophylaxis		
			Bacteriocidic solutions for daily wound care		
	Beginning of Surgical wound infection^uni^	Rise in H2	Meticulous wound care in the 1st week postop.		
	Epiglottoplasty^uni^	Rise in H3	Diminished use of this technique		
H3	Malnutrition (low proteins, alcohol abuse)^uni^	Rise in H3	Clinical nutritionist counselling	2014	L3
			Running sutures to close the neopharynx		
H4	Malnutrition (weight loss, low proteins)^uni^	Rise in H4	Counsel of clinical nutritionist		
	Salvage total laryngectomy^bin^	Rise in H4	Proper flap selection	2023	Future control
	Surgical wound infection^bin^	Rise in H4	Further improvements		

## Data Availability

The data presented in this study are available on request from the corresponding author. The da-ta are not publicly available due to privacy matters.

## References

[1] Deniz M., Ciftci Z., Gultekin E. (2015). Pharyngoesophageal Suturing Technique May Decrease the Incidence of Pharyngocutaneous Fistula Following Total Laryngectomy. Surg. Res. Pract..

[2] Walton B., Vellucci J., Patel P.B., Jennings K., McCammon S., Underbrink M.P. (2018). Post-Laryngectomy Stricture and Pharyngocutaneous Fistula: Review of Techniques in Primary Pharyngeal Reconstruction in Laryngectomy. Clin. Otolaryngol..

[3] Kim D.H., Kim S.W., Hwang S.H. (2022). Predictive Value of Risk Factors for Pharyngocutaneous Fistula after Total Laryngectomy. Laryngoscope.

[4] Wang M., Xun Y., Wang K., Lu L., Yu A., Guan B., Yu C. (2020). Risk Factors of Pharyngocutaneous Fistula after Total Laryngectomy: A Systematic Review and Meta-Analysis. Eur. Arch. Otorhinolaryngol..

[5] Sayles M., Koonce S.L., Harrison L., Beasley N., McRae A.R., Grant D.G. (2014). Pharyngo-Cutaneous Fistula Complicating Laryngectomy in the Chemo-Radiotherapy Organ-Preservation Epoch. Eur. Arch. Otorhinolaryngol.

[6] Dedivitis R.A., Aires F.T., Cernea C.R., Brandão L.G. (2015). Pharyngocutaneous Fistula after Total Laryngectomy: Systematic Review of Risk Factors. Head Neck.

[7] Cavalot A.L., Gervasio C.F., Nazionale G., Albera R., Bussi M., Staffieri A., Ferrero V., Cortesina G. (2000). Pharyngocutaneous Fistula as a Complication of Total Laryngectomy: Review of the Literature and Analysis of Case Records. Otolaryngol. Head Neck Surg..

[8] Herranz J., Sarandeses A., Fernández M.F., Barro C.V., Vidal J.M., Gavilán J. (2000). Complications after Total Laryngectomy in Nonradiated Laryngeal and Hypopharyngeal Carcinomas. Otolaryngol. Head Neck Surg..

[9] Liang J.-W., Li Z.-D., Li S.-C., Fang F.-Q., Zhao Y.-J., Li Y.-G. (2015). Pharyngocutaneous Fistula after Total Laryngectomy: A Systematic Review and Meta-Analysis of Risk Factors. Auris Nasus Larynx.

[10] Gonzalez-Orús Álvarez-Morujo R., Martinez Pascual P., Tucciarone M., Fernández Fernández M., Souviron Encabo R., Martinez Guirado T. (2020). Salvage Total Laryngectomy: Is a Flap Necessary?. Braz. J. Otorhinolaryngol..

[11] Paydarfar J.A., Birkmeyer N.J. (2006). Complications in Head and Neck Surgery: A Meta-Analysis of Postlaryngectomy Pharyngocutaneous Fistula. Arch. Otolaryngol. Head Neck Surg..

[12] Weber R.S., Berkey B.A., Forastiere A., Cooper J., Maor M., Goepfert H., Morrison W., Glisson B., Trotti A., Ridge J.A. (2003). Outcome of Salvage Total Laryngectomy Following Organ Preservation Therapy: The Radiation Therapy Oncology Group Trial 91-11. Arch. Otolaryngol. Head Neck Surg..

[13] Šifrer R., Strojan P., Tancer I., Dolenc M., Fugina S., Zore S.B., Aničin A. (2023). The Incidence and the Risk Factors for Pharyngocutaneous Fistula following Primary and Salvage Total Laryngectomy. Cancers.

[14] Brierley J.D., Gospodarowicz M.K., Wittekin C. (2016). TNM Classification of Malignant Tumours.

[15] Tabet J.C., Johnson J.T. (1990). Wound Infection in Head and Neck Surgery: Prophylaxis, Etiology and Management. J. Otolaryngol..

[16] Simo R., French G. (2006). The Use of Prophylactic Antibiotics in Head and Neck Oncolog-ical Surgery. Curr. Opin. Otolaryngol. Head Neck Surg..

[17] Schwartz S.R., Yueh B., Maynard C., Daley J., Henderson W., Khuri S.F. (2004). Pre-dictors of Wound Complications after Laryngectomy: A Study of over 2000 Patients. Otolaryngol. Head Neck Surg..

[18] González-Márquez R., Rodrigo J.P., Suárez Nieto C. (2012). Prognostic Significance of Postoperative Wound Infections after Total Laryngectomy. Head Neck.

[19] Harris R., Ofo E., Cope D., Nixon I., Oakley R., Jeannon J.-P., Simo R. (2015). Current Trends in Antibiotic Prophylaxis for Laryngectomy in the UK—A National Survey. J. Laryngol. Otol..

[20] Cannon R.B., Houlton J.J., Mendez E., Futran N.D. (2017). Methods to Reduce Postop-erative Surgical Site Infections after Head and Neck Oncology Surgery. Lancet Oncol..

[21] Boscolo-Rizzo P., De Cillis G., Marchiori C., Carpenè S., Da Mosto M.C. (2008). Multivariate Analysis of Risk Factors for Pharyngocutaneous Fistula after Total Laryngectomy. Eur. Arch. Otorhinolaryngol..

[22] Wakisaka N., Murono S., Kondo S., Furukawa M., Yoshizaki T. (2008). Post-Operative Pharyngocutaneous Fistula after Laryngectomy. Auris Nasus Larynx.

[23] Palomar-Asenjo V., Sarroca Capell E., Tobías Gómez S., Pérez Hernández I., Palomar-García V. (2008). Pharyngocutaneous Fistula Following Total Laryngectomy. A Case-Control Study of Risk Factors Implicated in its Onset. Acta. Otorrinolaringol. Esp..

[24] Šifrer R., Aničin A., Pohar M.P., Žargi M., Pukl P., Soklič T., Strojan P. (2016). Pharyngocutaneous Fistula: The Incidence and the Risk Factors. Eur. Arch. Otorhinolaryngol..

[25] Aničin A., Šifrer R., Strojan P. (2015). Pectoralis Major Myocutaneous Flap in Primary and Salvage Head and Neck Cancer Surgery. J. Oral. Maxillofac. Surg..

[26] Weinberger J.M., Eliashar R., Hirshoren N. (2023). Classification of Postlaryngectomy Pharyngocutaneous Fistulae. Ear. Nose Throat J..

[27] Casasayas M., Sansa A., García-Lorenzo J., Venegas M.D.P., Quer M., León X. (2022). Pharyngocutaneous fistula in irradiated patients: Systematic review and our experience. J. Laryngol. Otol..

[28] Kushida-Contreras B.H., Manrique O.J., Gaxiola-García M.A. (2021). Head and Neck Reconstruction of the Vessel-Depleted Neck: A Systematic Review of the Literature. Ann. Surg. Oncol..

[29] Breik O., Praveen P., Parmar S. (2020). The vessel-depleted neck in head and neck microvascular reconstruction: Extreme solutions for extreme situations. Curr. Opin Otolaryngol. Head Neck Surg..

[30] Prince A.D.P., Broderick M.T., Neal M.E.H., Spector M.E. (2020). Head and Neck Reconstruction in the Vessel Depleted Neck. Front. Oral. Maxillofac. Med..

[31] Piazza C., Taglietti V., Nicolai P. (2012). Reconstructive options after total laryngectomy with subtotal or circumferential hypopharyngectomy and cervical esophagectomy. Curr. Opin. Otolaryngol. Head Neck Surg..

[32] Costantino A., Festa B.M., Kim S.H., Baik F.M., Wang C.C., Pirola F., Malvezzi L., Spriano G., Mercante G., De Virgilio A. (2023). Complications of pectoralis major myo-cutaneous flap, anterolateral thigh flap and radial forearm free flap after total laryngectomy with partial pharyngectomy: A systematic review and network meta-analysis. Microsurgery.

[33] Rao K.N., Arora R.D., Singh A., Nagarkar N.M., Aggarwal A. (2022). Pharyngocutaneous Fistula Following Primary Total Laryngectomy: A Meta-analysis. Indian J. Surg. Oncol..

[34] Cecatto S.B., Soares M.M., Henriques T., Monteiro E., Moura C.I. (2014). Predictive factors for the postlaryngectomy pharyngocutaneous fistula development: Systematic review. Braz. J. Otorhinolaryngol..

